# Reduced Retinal Pigment Epithelial Autophagy Due to Loss of Rab12 Prenylation in a Human iPSC-RPE Model of Choroideremia

**DOI:** 10.3390/cells13121068

**Published:** 2024-06-19

**Authors:** Maide Ö. Raeker, Nirosha D. Perera, Athanasios J. Karoukis, Lisheng Chen, Kecia L. Feathers, Robin R. Ali, Debra A. Thompson, Abigail T. Fahim

**Affiliations:** 1 Department of Ophthalmology, University of Michigan, Ann Arbor, MI 48105, USA; mraeker@med.umich.edu (M.Ö.R.); nperera@med.umich.edu (N.D.P.); akarouki@med.umich.edu (A.J.K.); keciaf@med.umich.edu (K.L.F.); robin.ali@kcl.ac.uk (R.R.A.); dathom@med.umich.edu (D.A.T.); 2 Department of Orthopedic Surgery, University of Michigan, Ann Arbor, MI 48109, USA; lishengc@med.umich.edu; 3 KCL Center for Cell and Gene Therapy, London WC2R 2LS, UK; 4 Department of Biological Chemistry, University of Michigan, Ann Arbor, MI 48109, USA

**Keywords:** human pluripotent stem cells, disease modeling, retinal pigment epithelium, inherited retinal disease, retinal degeneration, choroideremia, autophagy

## Abstract

Choroideremia is an X-linked chorioretinal dystrophy caused by mutations in *CHM*, encoding Rab escort protein 1 (REP-1), leading to under-prenylation of Rab GTPases (Rabs). Despite ubiquitous expression of *CHM*, the phenotype is limited to degeneration of the retina, retinal pigment epithelium (RPE), and choroid, with evidence for primary pathology in RPE cells. However, the spectrum of under-prenylated Rabs in RPE cells and how they contribute to RPE dysfunction remain unknown. A CRISPR/Cas-9-edited *CHM^−/−^* iPSC-RPE model was generated with isogenic control cells. Unprenylated Rabs were biotinylated in vitro and identified by tandem mass tag (TMT) spectrometry. Rab12 was one of the least prenylated and has an established role in suppressing mTORC1 signaling and promoting autophagy. *CHM^−/−^* iPSC-RPE cells demonstrated increased mTORC1 signaling and reduced autophagic flux, consistent with Rab12 dysfunction. Autophagic flux was rescued in *CHM^−/−^* cells by transduction with gene replacement (ShH10-CMV-*CHM*) and was reduced in control cells by siRNA knockdown of Rab12. This study supports Rab12 under-prenylation as an important cause of RPE cell dysfunction in choroideremia and highlights increased mTORC1 and reduced autophagy as potential disease pathways for further investigation.

## 1. Introduction

Choroideremia affects approximately 1 in 50,000 people and is the most common inherited chorioretinal dystrophy, a group of disorders causing severe atrophy of the choroid in addition to the retinal pigment epithelial (RPE) cells and photoreceptors. There are no treatments for choroideremia, and patients are frequently legally blind by early adulthood [1]. Multiple lines of evidence implicate primary RPE cell pathology. Patient histology samples show widespread RPE cell irregularity in size and pigmentation, even in areas of histologically normal photoreceptors and choroid [2,3]. In vivo adaptive optics studies similarly show RPE cell hypertrophy relative to photoreceptors and choroidal endothelial cells, and near-infrared autofluorescence shows diffusely reduced signal from RPE melanin, even in areas of preserved retina [4,5]. Tissue-specific knockout mice and mosaic zebrafish demonstrate that primary RPE pathology contributes to retinal degeneration, although primary photoreceptor degeneration also occurs in the mouse model [6,7].

Choroideremia is caused by loss-of-function mutations in *CHM*, which encodes Rab escort protein 1 (REP-1), required as a chaperone for prenylation of Rab GTPases (Rabs) by geranylgeranyl transferase II (a.k.a. Rab GGTase) [8,9]. Dual prenyl groups are added to Rabs on two C-terminal cysteines, most commonly in a CC or CXC motif, and dual prenylation is required for Rabs to associate with lipid membranes and correctly direct protein trafficking throughout the cell and for extracellular export [10,11]. Under-prenylation of Rabs has been demonstrated in multiple models of choroideremia, yet it remains unknown how this leads to RPE dysfunction and chorioretinal degeneration [9,12,13,14,15,16]. The homologous REP-2, encoded by *CHML*, shares redundant function with REP-1, and hypotheses to explain the eye-exclusive phenotype in choroideremia have included the possibility of specific Rabs with particular importance in the eye that have less efficient prenylation with REP-2, or higher REP-2 expression compensating in non-ocular tissues [17]. Two prior studies have used proteomics approaches to comprehensively characterize the most under-prenylated Rabs resulting from prenylation deficiency, with overlapping but variable sets of Rab proteins identified [15,16]. In one study using pharmacologic prenylation inhibition in HeLa cells and then measuring the efficiency of Rab prenylation in an in vitro assay, Rabs38, 27a, 27b, 42, and 3d were the top five Rabs with the slowest prenylation [15]. In the second study using Rep-1 knockout in mouse embryonic fibroblasts, Rabs12, 27b, 5b, 21, and 24 were the top five unprenylated Rabs [16]. Additionally, in both studies, Rab5c and Rab23 were in the top 10. These studies were performed in non-RPE models. Since the proposed hypotheses to explain the phenotype are contingent on RPE-specific features, investigation in an RPE model is a critical next step to identify the Rabs most relevant to this disease.

A recent gene therapy trial using subretinal delivery of AAV-packaged *CHM* for gene augmentation in choroideremia failed to meet its primary endpoint, which was an improvement in the visual acuity of 15 letters (NCT03496012). Though gene therapy may hold great promise for choroideremia and other chorioretinal dystrophies, the slow path forward and lack of approved treatments highlight the need to pursue other strategies in parallel. We propose that identifying specific under-prenylated Rabs and defects in RPE function resulting from Rab prenylation deficiency will advance our understanding of the disease and reveal therapeutic targets. In this study, we report the generation of a CRISPR-edited *CHM^−/−^* iPSC-RPE model of choroideremia with isogenic control cells and demonstrate the spectrum of unprenylated Rabs. Furthermore, *CHM^−/−^* iPSC-RPE cells demonstrate increased mTORC1 signaling and reduced autophagy, which are known downstream targets of Rab12, one of the most unprenylated Rabs in our model.

Increased mTORC1 signaling and impaired RPE cell autophagy contribute to other forms of retinal degeneration, including age-related macular degeneration (AMD), Stargardt Disease, and PROM1-associated macular dystrophy [18,19,20,21,22]. Autophagy is a cellular catabolic process compensating for nutrient deprivation by lysosomal digest of intracellular damaged proteins and organelles [18]. Nutrient-rich conditions stimulate mTORC1 signaling, which promotes protein synthesis and inhibits autophagy. Lysosomal dysfunction has been previously described in models of choroideremia, and therefore, our findings in this report complement and build upon these prior studies to further elucidate how loss of Rab prenylation leads to RPE cell dysfunction in choroideremia [14,23].

## 2. Materials and Methods

### 2.1. CRISPR Gene Editing

Human iPSCs derived from healthy donor skin fibroblasts were purchased from LAgen Laboratories LLC (Rochester, MN, USA, Cat# 006-BIOTR-0001 clone 1). This study was approved by the University of Michigan Human Pluripotent Stem Cell Research Oversight committee (HPSCRO, Ann Arbor, MI, USA). Gene editing of the iPSC line was performed in the Human Stem Cell and Gene Editing Core (HSCGE) at the University of Michigan. iPSCs were cultured on a 6-well plate. The sgRNA targeting exon 1 of *CHM* (sequence ACGATCACATCAAACTCCGA) was cloned into PX458. One microgram of sgRNA-Cas9 DNA was transfected into 1 × 10^6^ iPS cells, and after 3 days, GFP-positive cells were sorted by fluorescence-activated cell sorting (FACS) and collected at 1 cell/well in 96-well plates. The genomic DNA was purified and analyzed from 25 expanded clonal cell populations. A second round of FACS and DNA analysis was performed to obtain double-knockout cell lines. The knockout cell line was confirmed by Sanger sequencing and next-generation deep sequencing to exclude off-target effects.

### 2.2. Cell Culture

*CHM^−/−^* and isogenic control iPSCs were cultured on 6-well plates in mTeSR^TM^ 1 media (STEMCELL TECHNOLOGIES, Seattle, WA, USA, Cat# 85850). iPSCs were differentiated into RPE as previously described and as shown in Figure S1 [24]. iPSCs were passaged and grown to confluency. After three days of confluency, media was changed from mTeSR^TM^ 1 to retinal differentiation media (RDM: 68% DMEM (Life Technologies, Carlsbad, CA, USA, Cat# 11965-092), 29% F12 (Life Technologies Cat# 31765-027), 1% penicillin/streptomycin (Life Technologies Cat# 15240-062), 2% B27 without retinoic acid (Gibco, Waltham, MA, USA, Cat# 12587010)). Between day 50 and day 90, colonies of RPE (with appropriate pigment and morphology) were dissected using a 21-gauge needle and passaged onto a 24-well laminin-coated plate at 50,000 cells/well. By 5 weeks, cells were pigmented and forming blebs, indicating polarization. Cells were maintained on plastic for 4–6 months and then passaged onto laminin-coated Transwell^®^-Clear Insert Polyester (PET) membranes (Corning, Corning, NY, USA, Cat# 3470) at a density of 335,000 cells/cm^2^ 2 months before they were needed for experiments. Fetal bovine serum (FBS) was added to retinal differentiation media (RDM) at 10% for 1 week, 3% for 1 week, and 2% for 1 week. Transepithelial electrical resistance (TEER) is a measure of RPE cell polarity and overall health and was used as a quality control measure. TEER was measured once weekly starting at 4 weeks post-passage, using an EVOM device with an STX2 electrode (World Precision Instruments, Sarasota, FL, USA). iPSC-RPE cells were be maintained on plastic for many months without changing pigmentation and morphology and without compromising their ability to form polarized RPE monolayers on Transwell supports when passaged, with high TEER in the range of 400–500 ohm × cm^2^. Therefore, cells were maintained on plastic as a ready supply and passaged onto Transwell supports 2 months before they were needed for upcoming experiments. Age-matched cells (i.e., same length of time on laminin-coated plastic and on Transwell permeable supports) were used for all experiments. For all subsequent experiments, except for the mass spectrometry studies (as detailed below), iPSC-RPE cells were used 2 months after passage onto Transwell supports.

For generating larger cell cultures for mass spectrometry studies, a higher-yield serial passage protocol without needle dissection was used to differentiate RPE cells [25]. When iPSCs reached 60–70% confluency, cells were treated with 5 µm Blebbistatin (Millipore Sigma, Burlington, MA, USA, Cat# B0560) in mTeSR^TM^ 1 for one hour, then passaged using Accutase to separate cell clumps. Single cells were seeded at a density of 20,000 cells/cm^2^ in mTeSR^TM^ 1 media + Blebbistatin onto plates coated in Geltrex (ThemoFisher/Gibco, Waltham, MA, USA, Cat#A1413301). After 48 h, media was changed to mTeSR^TM^ 1 (without Blebbistatin). At day 50, cells were treated with Collagenase IV (Life Technologies Corporation, Carlsbad, CA, USA, Cat# 17104019) for 4 h. The loosened monolayer was collected and vigorously pipetted up and down to break down clumps. Cells were collected by centrifugation at 350× *g* for 5 min, resuspended in Accutase (Sigma Aldrich, Saint Louis, MO, USA, Cat# A6964), and incubated at 37 °C for 20–25 min. After achieving mostly single cells, the enzymatic reaction was stopped by media dilution. The cell suspension was passed through a 40 µm nylon mesh, then centrifuged at 350× *g*. The resultant pellet was resuspended in RDM and plated at a density of 100,000 cells/cm^2^ on Synthemax II-SC Substrate (Corning, Corning, NY, USA, Cat#3535XX1)-coated plates. Fifteen days after P1, cells were passaged again (P2) and were used for experiments at least 40 days post-passage.

### 2.3. AAV Transduction

The human *CHM* cDNA (NM_000390.4) was cloned into an AAV viral genomic plasmid under the control of the CMV promoter (pD10-Pst2), and the insert was confirmed by restriction digest. The plasmid was packaged into ShH10 (a serotype of AAV2) at Virovek (Hayward, CA, USA). RPE cells were transduced using a viral multiplicity of infection (MOI) of 100,000. The ShH10-CMV-CHM was diluted 1:10 with pluronic Poloxamer 188 Non-ionic Surfactant (100X) (F-68) (ThermoFisher/Gibco, Waltham, MA, USA, Cat# 24040-032) and incubated for 1 h at room temperature. The F-68/viral dilution was added to RDM and applied to the apical chamber of the RPE cells on Transwells. The next day, additional RDM was added, after which media changes proceeded per the usual schedule.

### 2.4. In Vitro Prenylation and Mass Spectrometry

iPSC-RPE cells maintained on 6-well plates were treated with either DMSO (Sigma, Saint Louis, MO, USA, Cat# D2650) or 10 μM compactin (Cayman Chemical, Ann Arbor, MI, USA, Cat # 10010340) for 24 h, 9 wells per experimental group. Cells were then lysed with prenylation/lysis buffer as described, with the following modifications [26]. Cells were washed three times with ice-cold phosphate-buffered saline (PBS) (ThermoFisher/Gibco, Waltham, MA, USA, Cat# 14040-133), then harvested in 1 mL ice-cold prenylation/lysis buffer using a cell scraper. Lysates were collected into 15 mL conical tubes and incubated on ice for 30 min, with gentle vortexing every 10 min. Cells were then homogenized with a handheld homogenizer for 20 cycles, followed by passing the cells through a 25-gauge needle 15 times. Homogenized cells were centrifuged at 1500× *g* for 5 min at 4 °C, and the supernatant was transferred to a fresh tube and centrifuged at 100,000× *g* at 4 °C for 1 h. The supernatant was collected and concentrated using Ultra-2 Centrifugal Concentrator/Filter Unit, 3 kDa MWCO (Sigma, Saint Louis, MO, USA, Cat# UFC200324) at 3500× *g*. Protein concentration was determined using a BIO-RAD DC protein assay (BIO-RAD, Hercules, CA, USA).

The prenylation reactions for mass spectrometry were set up using 2.4 mg of cell lysate (prepared as described above) in prenylation buffer containing 20 µM guanosine 5′-diphosphate (GDP) (Sigma-Aldrich, Saint Louis, MO, USA, Cat# G7217), 2 µM purified REP-1 (Jena Bioscience, Jena, Germany/Sapphire North America, Ann Arbor, MI, USA, Cat# PR-105), 2 µM geranylgeranyltransferase type II (GGTase-II) (Jena Bioscience, Jena, Germany/Sapphire North America, Ann Arbor, MI, USA, Cat# PR-103), 5 µM biotin-labeled geranyl pyrophosphate, triethylammonium salt (B-GPP) (Jena Bioscience, Jena, Germany/Sapphire North America, Ann Arbor, MI, USA, Cat# Li-015), and 2 mM dithiothreitol (DTT) (ThermoFisher, Waltham, MA, USA, Cat# A39255). The reactions were incubated for 2 h at 37 °C. Experiments were performed in triplicate.

Prenylated lysates were incubated with magnetic streptavidin beads (New England Biolabs, Ipswich, MA, USA, Cat# S1420S) overnight at 4 °C to pull down the biotin-tagged proteins. Protein-bound beads were washed with RIPA buffer (ThermoFisher, Waltham, MA, USA, Cat# 89900) three times and collected in PBS. Recoverin was diluted in the samples to equal concentration for normalization. Tandem mass tag (TMT) labeling and LC–MS/MS analysis was performed at the University of Michigan Proteomics Resource Facility using TMT-10 plex (ThermoFisher, Waltham, MA, USA, Cat# 90110) and an Orbitrap Tribid Fusion mass spectrometer (ThermoFisher, Waltham, MA, USA) as previously described [24]. The data were analyzed using Proteome Discoverer (Version 2.1, Thermo Fisher, Waltham, MA, USA). Signal-to-noise values for each reporter ion in the MS/MS data were extracted and summed for each peptide, normalized based on recoverin S/N, and scaled to a total intensity of 100% across channels. MS/MS spectra were searched against the UniProt human protein database [27]. Pathway analysis was performed in iPathwayGuide (Advaita Bioinformatics, Ann Arbor, MI, USA) [28].

Cell lysates from *CHM^−/−^* and isogenic control iPSC-RPE cells were also prepared for western blot experiments at a smaller scale. In vitro prenylation reactions were set up as above but using 50 ug of proteins in 40 µL prenylation reactions. The reactions were ended with sample buffer and analyzed by SDS-PAGE and western blotting. Incorporation of biotinylated lipid donor into unprenylated Rabs was detected with streptavidin-HRP (1:1000 dilution, Invitrogen, Waltham, MA, USA, Cat# 434323, Lot# WA315882).

### 2.5. Cell Fractionation

Subcellular fractionation of RPE cells was performed by ultracentrifugation as previously described, with the following modifications [13]. RPE cells grown in 6-well plates were washed 3 times with ice-cold PBS, scraped into ice-cold PBS containing protease inhibitor cocktail (ThermoFisher Cat# 87785), transferred into microcentrifuge tubes, and centrifuged at 400× *g* for 5 min at 4 °C. Pelleted cells were resuspended in fractionation lysis buffer (50 mM Tris-HCl (pH 7.5), 150 mM NaCl, 2 mM MgCl_2_, 0.5 mM EGTA, 0.5 mM EDTA, 5 mM DTT, 0.1 mM GDP, and 1X protease inhibitor cocktail) and incubated on ice for 15 min. Homogenization was completed by passing the cells through a 27-gauge needle 20 times using a 0.5 mL syringe, and cell debris was removed by centrifugation at 1500× *g* for 5 min at 4 °C. Post-nuclear supernatant was ultracentrifuged at 100,000× *g* for 1 h at 4 °C to separate cytosolic and membrane fractions. The supernatant (cytosolic fraction) was collected, and the pellet (membrane fraction) was resuspended in fractionation lysis buffer containing 1% Nonidet P-40 (ThermoFisher, Cat# 85124) to solubilize the membrane proteins to a total volume equal to the volume of the collected supernatant (cytosolic fraction). The membrane suspension was incubated on ice for 30 min, centrifuged at 100,000× *g* for 1 h at 4 °C, and resulting supernatant, containing detergent soluble membrane-associated proteins, was collected. Proper fractionation of the cytosol and membranes was validated by immunoblotting using antibodies against b-actin and Na^+^/K^+^ ATPase, respectively. Fractions were analyzed by SDS-PAGE and western blotting for Rab27a, Rab12, Rab8a, and Rab5a. Antibodies to β-actin and Na^+^/K^+^ ATPase were used as loading controls to normalize the amounts of Rab proteins in cytosolic and membrane fractions, respectively. The blots were imaged and analyzed using Azure c600 Imaging System and software (V13.2, Azure Biosystems, Dubin, CA, USA), respectively. Experiments were performed in triplicate. 

### 2.6. Western Blot

Samples were mixed with Laemmli sample buffer, boiled at 95 °C for 5 min, and analyzed by western blot. Proteins were separated via SDS–polyacrylamide electrophoresis (SDS/PAGE) using 4–15% Mini-PROTEAN TGX Precast Protein Gels (BIO-RAD, Hercules, CA, USA) and transferred onto PVDF membranes using a Trans-Blot Turbo System (BIO-RAD). The membranes were blocked in SuperBlock T20 blocking buffer (ThermoFisher Cat# 37536) for 1 h at room temperature, incubated with primary antibody overnight, washed, and incubated with secondary antibody for 1 h at room temperature. Blots were developed with EcoBright Femto HRP (Innovative Solutions, Henrietta, NY, USA, Cat# EBFH100). Bands were visualized and photographed using an Azure c600 Imaging System (Azure Biosystems, Dublin, CA, USA). Densitometry quantification was performed using AzureSpot software (V13.2, Azure Biosystems, Dublin, CA, USA). Primary antibodies used were anti-REP1 (Santa Cruz, Dallas, TX, USA, Cat# sc-23905, Lot# K1920), streptavidin horseradish peroxidase (HRP) conjugate (Invitrogen 434323), anti-Rab27a (Proteintech, Rosemont, IL, USA, Cat# 17817-1-AP, Lot# 97971), anti-Rab12 (Proteintech, Rosemont, IL, USA, Cat# 18843-1-AP, Lot# 99593), anti-Rab5a (Cell Signaling, Danvers, MA, USA, Cat# 46449, Lot# 3), anti-Rab8a (Abcam, Waltham, MA, USA, Ca# AB188574, Lot# 1028937.1), anti-β-actin (Cell Signaling, Danvers, MA, USA, Cat# 3700, Lot# 21), anti-Na^+^/K^+^ ATPase (Santa Cruz Cat# sc-58628, Lot# C0322), anti-ULK1P555 (Cell Signaling Cat# 5869), anti-ULK1P757 (Cell Signaling Cat# 6888, Lot# 4 and 5), anti-pp70S6K (Cell Signaling Cat# 9205), and anti-p70S6K (Cell Signaling Cat# 2708). Secondary antibodies used were goat anti-rabbit IgG (HRP) (Abcam Cat# AB205718, Lot# 1036958-20) and goat anti-mouse IgG (HRP) (Millipore, Burlington, MA, USA, Cat# AB124P).

### 2.7. RT-PCR

iPSCs and iPSC-RPE cells were harvested, and RNA was extracted using the RNeasy Mini Kit (Qiagen, Germantown, MD, USA, Cat# 74104). SuperScript II reverse transcriptase (Invitrogen, Waltham, MA, USA, Cat# 18064014) was used to make complementary DNA, which was used the same day to perform multiplexed RT-coupled PCR with POWER SYBR^TM^ Green PCR Master Mix (Applied Biosystems, Foster City, CA, USA, Cat# A25742) in a Biorad iCycler to measure transcript levels of *CHM*, *CHML,* and *RAB12*. Each sample was run in triplicate. Relative transcript levels were normalized to *ACTB* for *RAB12* and to *GAPDH* for *CHM* and *CHML*. All primer sequences are shown in Table S1.

### 2.8. Immunocytochemistry

iPSC-RPE cells on Transwell permeable supports were washed with PBS, fixed with 4% paraformaldehyde for 15 min, washed again with PBS, and the polyester membranes with fixed cells were then excised from the Transwell plastic insert. The cells were then blocked with blocking buffer (3% bovine serum albumin (BSA), 0.3 M glycine, 0.15% Triton X-100, 1% donkey serum) for 1 h. Cells were incubated in primary anti-REP1 antibody (Santa Cruz, Dallas, TX, USA, Cat# sc-23905) overnight at 4 °C, washed, and incubated in secondary antibody (donkey anti-mouse IgG-Alexa fluor 488 (Jackson Immunoresearch, West Grove, PA, USA, 715-545-150, Lot# 120317)). Images were acquired with a Leica SP5 confocal microscope (Leica Microsystems, Wetzlar, Germany).

### 2.9. Autophagic Flux

RPE cells were trypsinized with 50 µL of 0.25% Trypsin for 15 min at 37 °C. Cells were passaged onto a 96-well plate in RDM and allowed to settle for 4 h at 37 °C. For mTOR inhibition samples, torin was added to the media at concentrations indicated in the figures for 24 h. For autophagy inhibition samples, 1 μM bafilomycin and 50 mM ammonium chloride were then added to the media for 90 min. LC3-II was then measured using LC3-II Quantitation Kit (Cell Biolabs, San Diego, CA, USA, Cat# CBA-5117), which utilizes a selective permeabilization procedure to remove the cytosolic pro-LC3 and LC3-I and retain the autophagosome membrane bound LC3-II. After fixation and permeabilization, cells were probed with an anti-LC3 A/B antibody, followed by an HRP-conjugated secondary antibody. After developing, the optical density of each well was determined using a microplate reader.

### 2.10. siRNA Knockdown

In order to achieve Rab12 knockdown, siRNA from Dharmacon was used. siRNA-Rab12 (Dharmacon, Lafayette, CO, USA, L-023375-02-005), siRNA-scramble control (D-001810-10-05), and D4 reagent (T-2004-01) were each diluted with antibiotic-free media and incubated for 5 min. The diluted D4 was added to the diluted siRNA-Rab12 and to the diluted siRNA-scramble and incubated at room temperature for an additional 20 min. The resulting master mixes of siRNA-Rab12/D4 and siRNA-scramble control/D4 were added to iPSC-RPE cells with an additional 80 μL of antibiotic-free medium per sample. Cells were incubated at 37 degrees and harvested at 48 h for RNA, at 72 h for protein (with media change at 48 h), or used for other assays as indicated. 

### 2.11. Rab12 Enzyme-Linked Immunosorbent Assay (ELISA)

Rab12 protein was measured using the human Ras-related protein Rab-12 (RAB12) ELISA Kit (Abbexa, Sugar Land, TX, USA, abx541064) according to the manufacturer’s protocol. Cells were trypsinized, centrifuged, and washed 3 times with ice-cold PBS and resuspended in PBS. Cells were lysed with ultra-sonification 4 times and centrifuged at 1500× *g* for 20 min. The supernatant was analyzed immediately. A standard was prepared with serial dilutions of recombinant Rab12. Standards and cell lysate samples were added to a pre-coated plate and incubated for 2 h at 37 °C. Detection reagent A was added to each well and incubated for 1 h at 37 °C. The plate was washed 3 times with wash buffer and then incubated with Detection reagent B for 1 h at 37 °C. The plate was washed 5 times with buffer, incubated with substrate for 20 min at 37 °C, and then stopped with Stop Solution and measured immediately at OD 450 nm using a microplate reader.

### 2.12. 4EBP-1pSer65 Immunoassay

Phosphorylated 4EBP1 was measured using the Lumit^®^ Immunoassay Cellular System for Phospho-4E-BP1(Ser65) (Promega, Madison, WI, USA, W1331) according to the manufacturer’s protocol. Briefly, cells were lysed by adding lysis buffer to the wells for a final dilution of 1:5 in media, and the plate was shaken at 800 rpm for 40 min. An antibody mixture containing 2 primary antibodies and 2 secondary antibodies diluted in immunoassay reaction buffer was added to each well. The 2 primary antibodies were mouse anti-phospho-4EBP1 (Thermo Fisher MA5-31843, Lot# ZD4292518) and rabbit anti-4E-BP1 (Cell Signaling Technology 9644, Lot# 13). The 2 secondary antibodies were Lumit^®^ anti-mouse antibody-LgBiT and Lumit^®^ anti-rabbit antibody-SmBiT. LgBiT and SmBiT are 2 subunits that form an active NanoLuc^®^ luciferase when they are brought into close proximity by binding to the same protein. Since each secondary antibody will only bind to one of the primary antibodies due to the different host species of the primary antibodies, active luciferase will only form when both primary antibodies bind the same protein (i.e., only phosphorylated 4EBP1). After adding the antibody mixture, the plate was shaken at 400 rpm for 2 min and incubated at room temperature for 90 min. Then, the Lumit^®^ Detection Reagent was added, the plate was shaken for 2 min at 400 rpm, and the luminescence was measured with a microplate reader.

### 2.13. Statistical Analysis

To compare Rab levels in compactin-treated cells vs. untreated cells in the TMT mass spectrometry experiments, *p*-values were calculated using Proteome Discoverer software (version 2.1, ThermoFisher, Waltham, MA, USA) using a Student’s *t*-test for each Rab (comparing levels between compactin-treated and untreated cells) and adjusted for multiple comparisons using the Benjamini–Hochberg false discovery rate approach. For other experiments, for simple comparisons, a Student’s *t*-test was used, or ANOVA was used for comparison of more than 2 means. Statistical tests used, sample sizes, and *p*-values are specified in each figure legend.

## 3. Results

### 3.1. CHM^−/−^ iPSC-RPE Cells Were Generated

**Figure 1:**
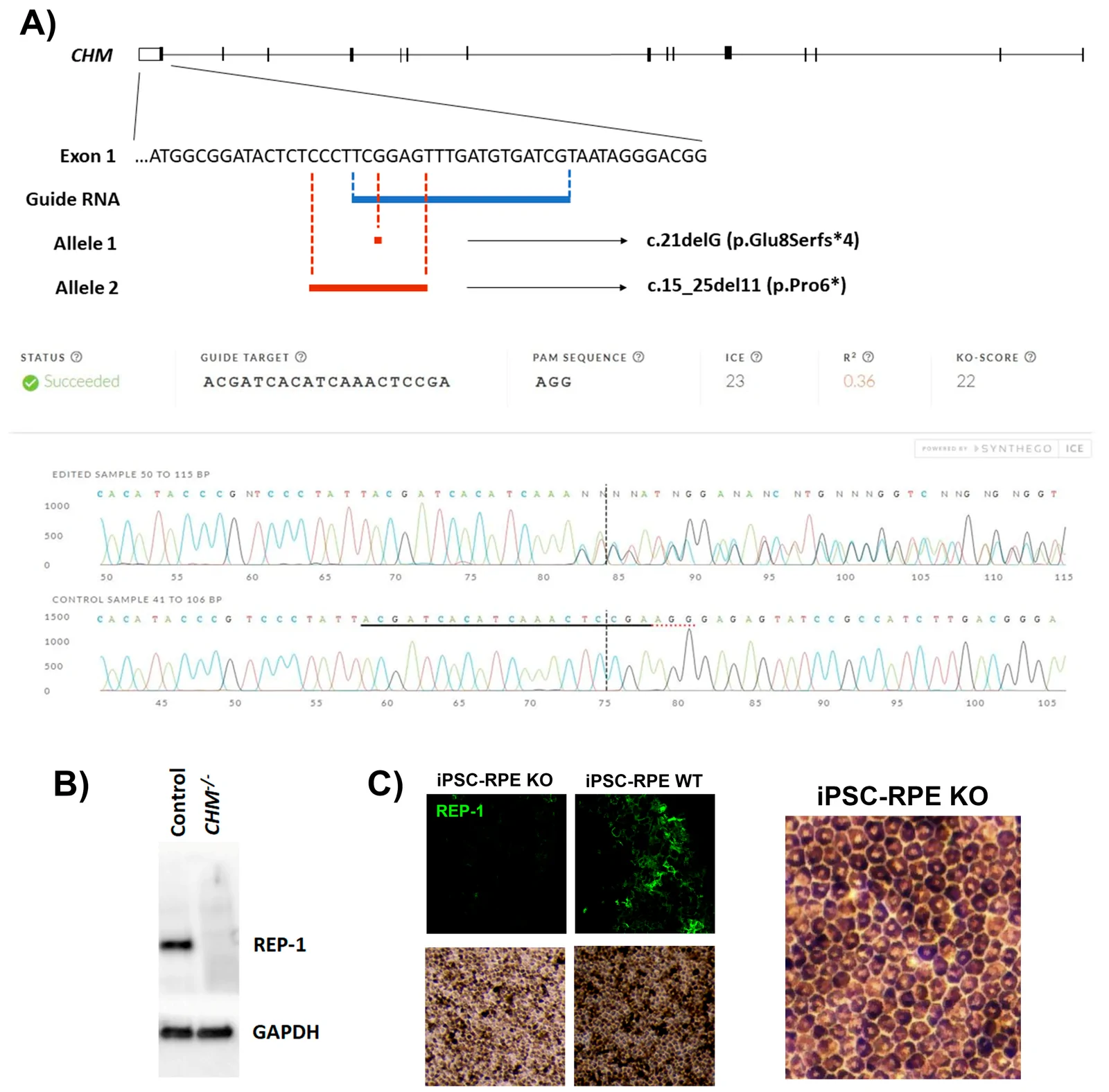
Generation of *CHM^−/−^* iPSC-RPE cells. (**A**) CRISPR/Cas-9 knockout of *CHM* targeting exon 1, resulting in biallelic variants with early stop codons (*). Sanger sequencing of the biallelic deletions is shown. (**B**) Western blot showing absence of Rab escort protein 1 in knockout (KO) iPS cells compared to wild-type (WT) iPS cells (representative blot shown; experiment was performed 3 times); and (**C**) KO and WT iPSC-RPE cells with immunocytochemistry (ICC) showing absence of Rab escort protein in KO cells (representative image shown; experiment was performed twice). A magnified image of iPSC-RPE cobblestone morphology is shown at bottom right and has been shown previously [24].

A validated human iPSC line generated from skin fibroblasts from a healthy control was obtained commercially. CRISPR/Cas-9 gene editing was used to generate biallelic variants with early stop codons in exon 1 of *CHM* in iPS cells (Figure 1A). RPE cells were differentiated from *CHM^−/−^* and isogenic control iPSCs. *CHM^−/−^* cells (both iPSCs and iPSC-RPE cells) had no detectable REP-1 protein (Figure 1B,C). *CHM^−/−^* iPSC-RPE cells demonstrated appropriate cobblestone morphology and melanin pigmentation (Figure 1C). Overall pigmentation was reduced in *CHM^−/−^* iPSC-RPE cells compared to control cells, although this was not quantified. RPE melanosome abnormalities have been reported previously in a mouse model of choroideremia [6].

### 3.2. CHM Is Upregulated, and CHML Downregulated, during RPE Differentiation

**Figure 2:**
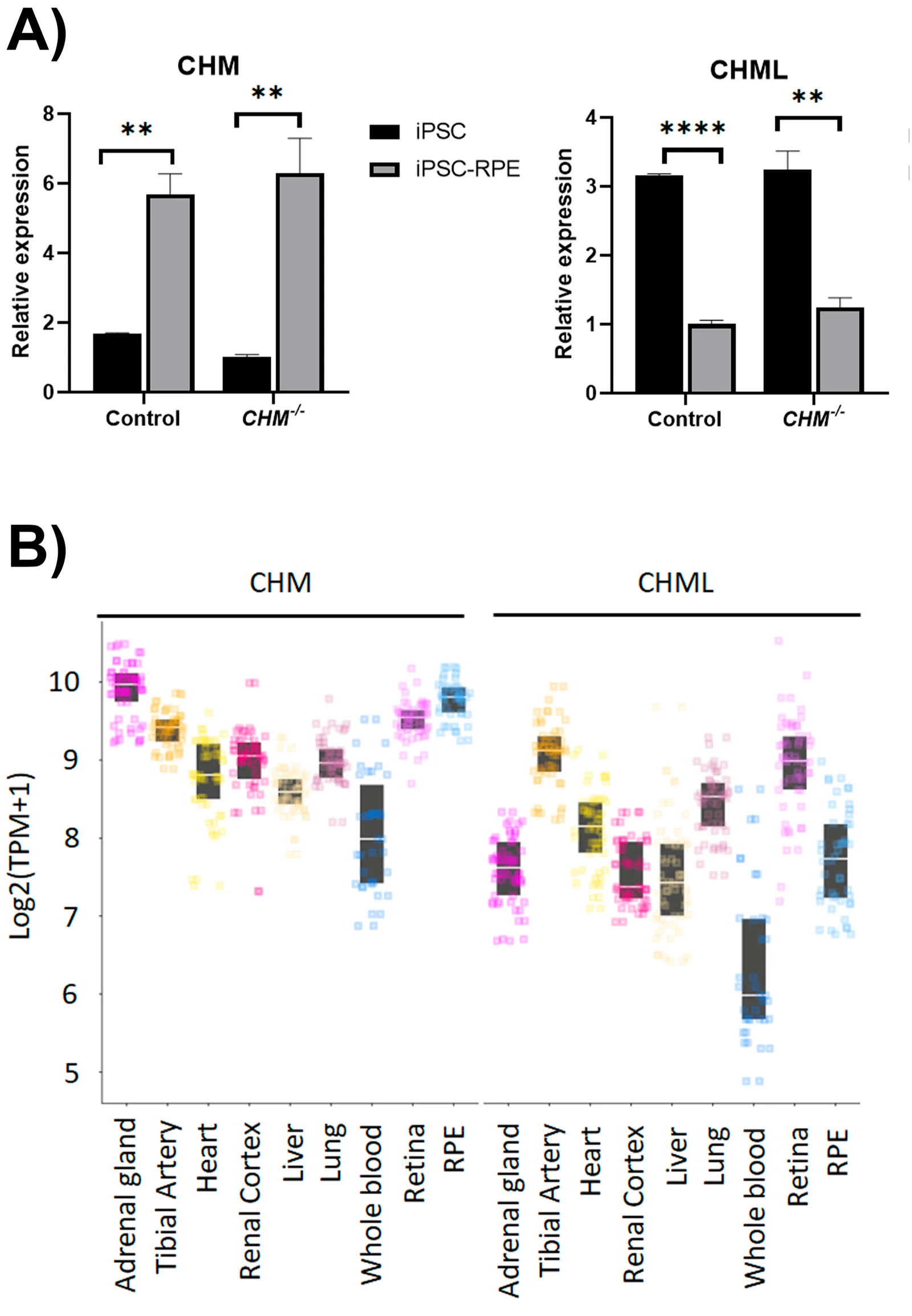
*CHM* and *CHML* expression. (**A**) Relative expression of *CHM* and *CHML* by quantitative RT-PCR shows that *CHM* increases during RPE differentiation, whereas *CHML* decreases, and expression is similar between control and *CHM^−/−^* cells, with no significant difference (n = 3, mean ± SD; Student’s *t*-tests ** *p* < 0.01, **** *p* < 0.0001; *CHM* RT-PCR was performed twice, and *CHML* RT-PCR was performed once). (**B**) Pooled RNAseq data from the NEI EyeIntegration project comparing transcript levels of *CHM* across human tissues and *CHML* across human tissues, including adult retina and adult RPE samples. The graph was generated at https://eyeintegration.nei.nih.gov (accessed on 10 May 2022) and was modified for legibility.

It has been hypothesized that relatively low expression of the homologous *CHML* gene in RPE cells could contribute to the eye-specific phenotype of choroideremia despite ubiquitous expression of *CHM*. *CHM* and *CHML* expression levels were therefore compared in iPSCs and mature iPSC-RPE cells. Both control and *CHM^−/−^* iPSC-RPE cells showed significantly higher *CHM* expression and significantly lower *CHML* expression than their pluripotent progenitors, suggesting that upregulation of *CHM* and downregulation of *CHML* is part of the RPE differentiation program (Figure 2A). The finding of *CHM* transcript in *CHM^−/−^* cells is consistent with previous reports that *CHM* escapes nonsense-mediated decay [29,30]. Despite the presence of *CHM* transcript in our cells, the absence of detectable REP-1 protein validated the knockout of *CHM* in our model (Figure 1B,C).

Although the downregulation of *CHML* in iPSC-RPE cells may suggest that low *CHML* expression could contribute to choroideremia, *CHML* expression in the RPE or retina is not necessarily lower than in other tissues. RNAseq data from the NEI EyeIntegration database suggest that *CHML* expression is roughly equivalent or higher in RPE and retina compared to other tissues, and *CHM* expression follows a similar pattern (Figure 2B) [31]. Therefore, the eye-exclusive phenotype in choroideremia may be more due to prenylation deficiency of Rabs with particular importance in RPE function, and therefore, defining the spectrum of unprenylated Rabs in *CHM^−/−^* iPSC-RPE and the downstream consequences of top candidates is the next step in elucidating the molecular mechanism of disease.

### 3.3. Thirty-Eight Unprenylated Rabs Were Identified in CHM^−/−^ iPSC-RPE Cells

**Figure 3:**
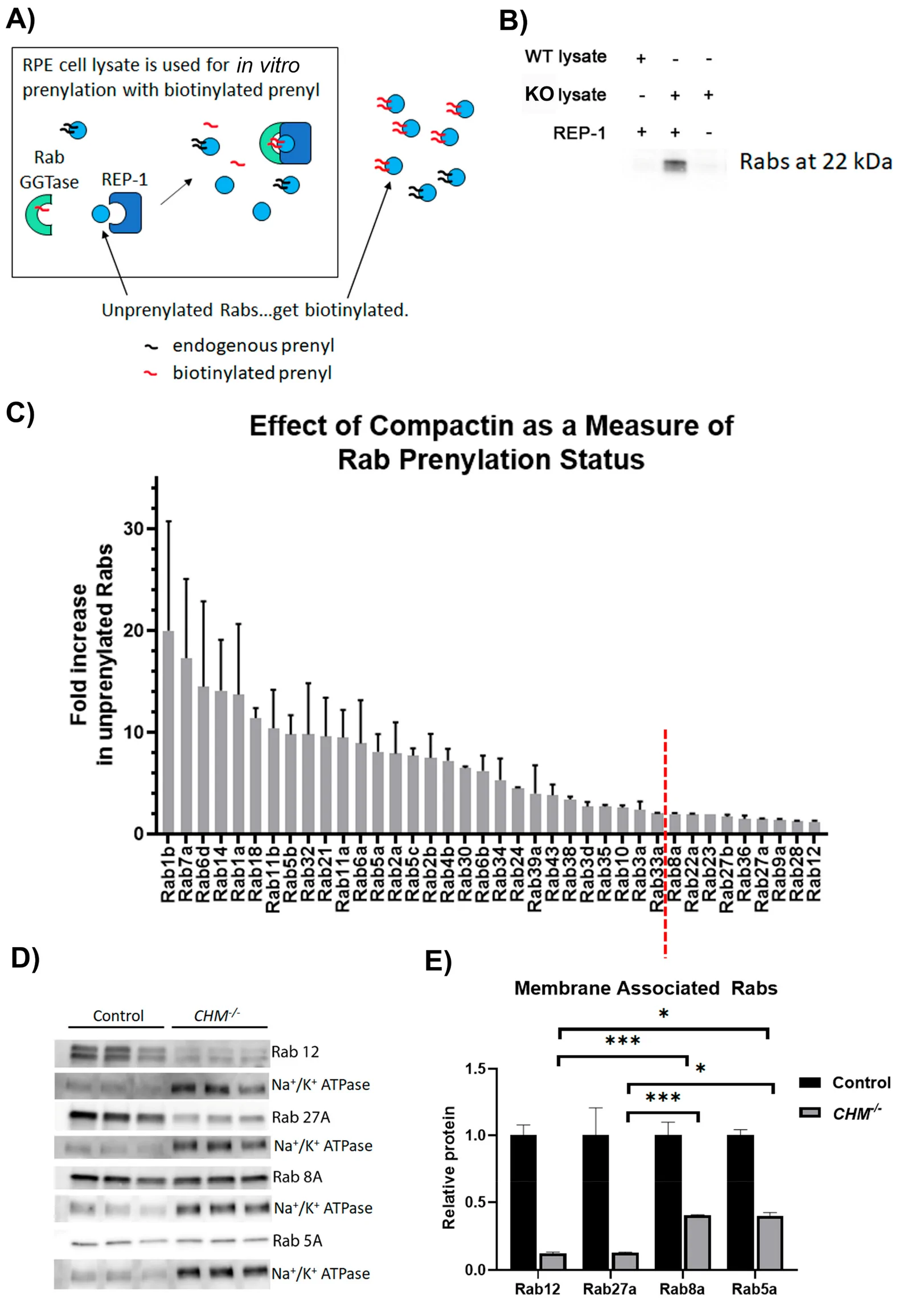
Thirty-eight unprenylated Rabs were identified in *CHM^−/−^* iPSC-RPE cells. (**A**) Schematic of the in vitro prenylation assay. (**B**) Western blot with HRP-labeled streptavidin shows biotinylated Rabs in the *CHM^−/−^* iPSC-RPE lysate when exogenous REP-1 is added compared to control lysate. This experiment was performed twice. (**C**) Graph showing the fold increase of each unprenylated Rab with compactin compared to baseline in *CHM^−/−^* iPSC-RPE lysate, as measured by TMT spectrometry. Dashed line indicates cut-off for *p* < 0.05 between compactin-treated and baseline to the left of the line. The TMT spectrometry was performed once. (**D**) Western blot of Rabs in the membrane-solubilized fraction of *CHM^−/−^* and control iPSC-RPE cells and Na^+^/K^+^ ATPase (a membrane protein) as a control. Due to the decreased levels of membrane-bound Rabs in *CHM^−/−^* cells, a higher sample volume was loaded to ensure Rabs could be detected and quantified by densitometry, which also resulted in darker Na^+^/K^+^ ATPase bands. All Rab densitometry was normalized to Na^+^/K^+^ ATPase, which had equal expression between *CHM^−/−^* and control cells (Figure S2). (**E**) Graph showing quantification of densitometry, normalized to Na^+^/K^+^ ATPase, as a proportion of control levels. N = 3, mean ± SEM, * *p* < 0.05, *** *p* < 0.001, experiment performed once.

Cell lysates from *CHM^−/−^* and isogenic control iPSC-RPE cells were used for in vitro prenylation with biotinylated prenyl groups to biotinylate unprenylated Rabs (Figure 3A). Western blot demonstrated a large band of biotinylated Rabs in *CHM^−/−^* iPSC-RPE cells compared to controls, confirming the Rab prenylation defect (Figure 3B). As there are over 70 known Rabs with different functions, a tandem mass tag (TMT)-plex spectrometry approach was used to determine the spectrum of unprenylated Rabs in *CHM^−/−^* iPSC-RPE cells. Compactin was used in cell culture to inhibit prenyl synthesis, thereby inhibiting all protein prenylation in an unbiased manner. By reducing the supply of prenyl groups, compactin is expected to have a greater impact on Rabs with otherwise good prenylation status at baseline and a smaller impact on Rabs with already poor prenylation at baseline in *CHM^−/−^* iPSC-RPE cells. In vitro prenylation with biotin-prenyl groups was performed, followed by streptavidin pull-down, and TMT spectrometry was used to compare the level of each unprenylated Rab with and without compactin treatment in *CHM^−/−^* iPSC-RPE cells (Figure 3C). Due to the efficient intracellular prenylation in control cells, the control cell lysate yielded almost no biotinylated Rabs, as seen in Figure 3B, and control samples were therefore not suitable for accurate comparisons in the TMT spectrometry analysis, which requires comparison of samples with similar protein concentration and heterogeneity. The TMT approach was used to compare compactin-treated to untreated *CHM^−/−^* iPSC-RPE cells to identify the most severely unprenylated Rabs in these cells, visualized by the dark band in Figure 3B. 

Thirty-eight unprenylated Rabs were identified, and 9 of the 38 had no statistically significant difference between the compactin-treated and untreated groups, suggesting poor prenylation at baseline that could not be significantly reduced further by inhibition of prenyl synthesis. Rab27a, which has been identified in multiple studies as poorly prenylated in choroideremia, was among the nine. To validate the mass spectrometry results and demonstrate that prenylation status was accurately approximated, we selected four Rabs for cell fractionation studies. Rab12, Rab27a, Rab8a, and Rab5a had fold-changes of 1.2, 1.5, 2.0, and 8.1, respectively, in unprenylated status with compactin treatment (Figure 3C). Membrane-bound proteins were separated using centrifugation and solubilization in both *CHM^−/−^* and control iPSC-RPE cells, followed by western blot for Rab12, Rab27a, Rab8a, and Rab5a (Figure 3D). Relative to control cells, *CHM^−/−^* iPSC-RPE cells had significantly less Rab12 or Rab27a in the membrane fraction (12% and 13% of control levels, respectively) compared to Rab8a or Rab5a (41% and 40% of control levels). These results may not represent exact quantification of membrane-bound Rabs due to the limitations of membrane solubilization and western blot as a quantitative method, but the results show the general trend that Rab 12 and Rab27a are less prenylated at baseline than Rab8a and Rab5a in *CHM^−/−^* iPSC-RPE cells, consistent with the mass spectrometry data.

### 3.4. mTORC1 Signaling Is Increased in CHM^−/−^ iPSC-RPE Cells

**Figure 4:**
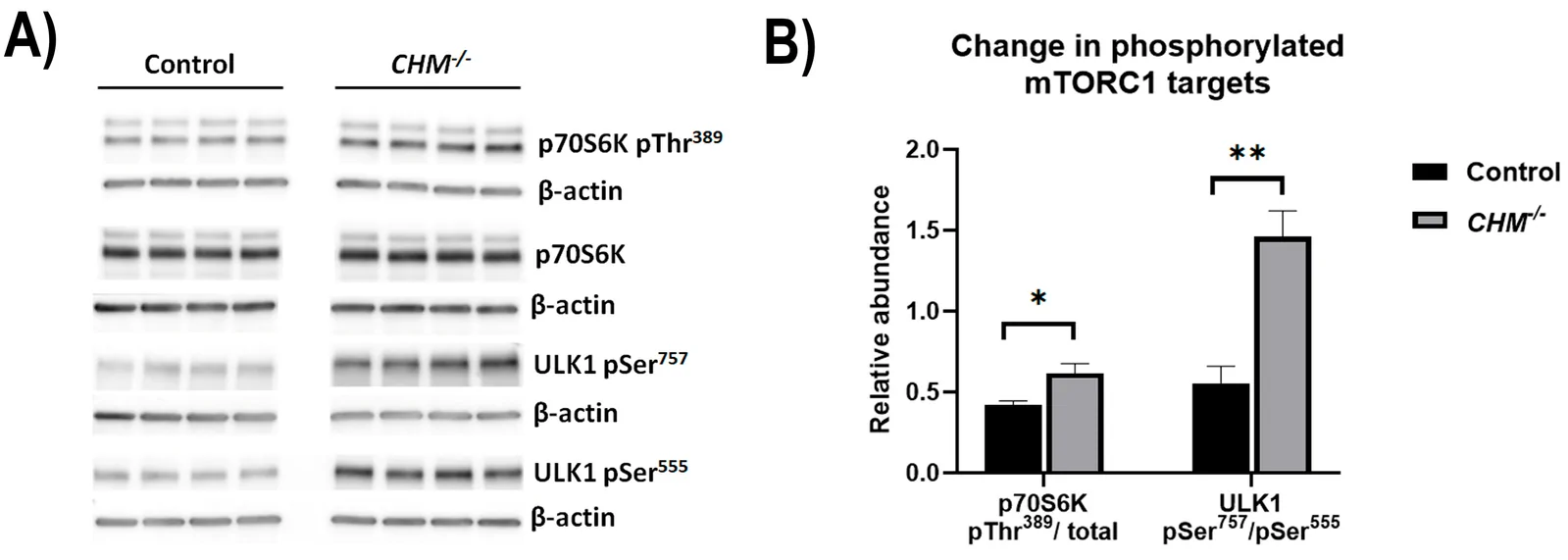
*CHM^−/−^* iPSC-RPE cells have increased mTORC1 signaling. (**A**) Western blot of downstream targets of mTORC1 phosphorylation in *CHM^−/−^* and control iPSC-RPE cells. P70S6K is phosphorylated on Thr 389 by mTORC1, and ULK1 is phosphorylated on Ser 757 by mTORC1 and phosphorylated on Ser 555 by AMPK when mTORC1 signaling is low. (**B**) Graph showing the ratio of phosphorylated to total p70S6K and the ratio of ULK1 phosphorylated on Ser 757 to ULK1 phosphorylated on Ser 555 as measures of mTORC1 activity in both *CHM^−/−^* and control iPSC-RPE cells. All densitometry was first normalized to β-actin. N = 4, mean ± SEM, analyzed by Student’s *t*-test, * *p* < 0.05, ** *p* < 0.01. This experiment was performed 3 times for ULK1 and twice for P70S6K.

Rab12 was one of the least prenylated Rabs in the *CHM^−/−^* iPSC-RPE cells and has a known role in promoting autophagy by regulating mTORC1 signaling [32]. We therefore investigated mTORC1 signaling in *CHM^−/−^* iPSC-RPE cells by measuring levels of phosphorylated downstream targets of mTORC1 (Figure 4). The p70 ribosomal protein S6 kinase (p70S6K) is a serine/threonine kinase that promotes protein synthesis and is activated by phosphorylation on threonine 389 by mTORC1 [33]. The ULK1 kinase promotes autophagy and is inhibited by mTORC1 via phosphorylation on serine 757, and it is activated by phosphorylation on several residues, including serine 555 by AMP-activated protein kinase (AMPK) [34]. The proportion of phosphorylated p70S6K (p70S6K pThr389/total) and the ratio of ULK1 phosphorylated on serine 757 to ULK1 phosphorylated on serine 555 (ULK1 pSer757/pSer555) were measured by western blot as measures of mTORC1 activity. Both were increased in *CHM^−/−^* iPSC-RPE cells (Figure 4A,B). 

### 3.5. Autophagic Flux Is Reduced in CHM^−/−^ iPSC-RPE Cells

**Figure 5:**
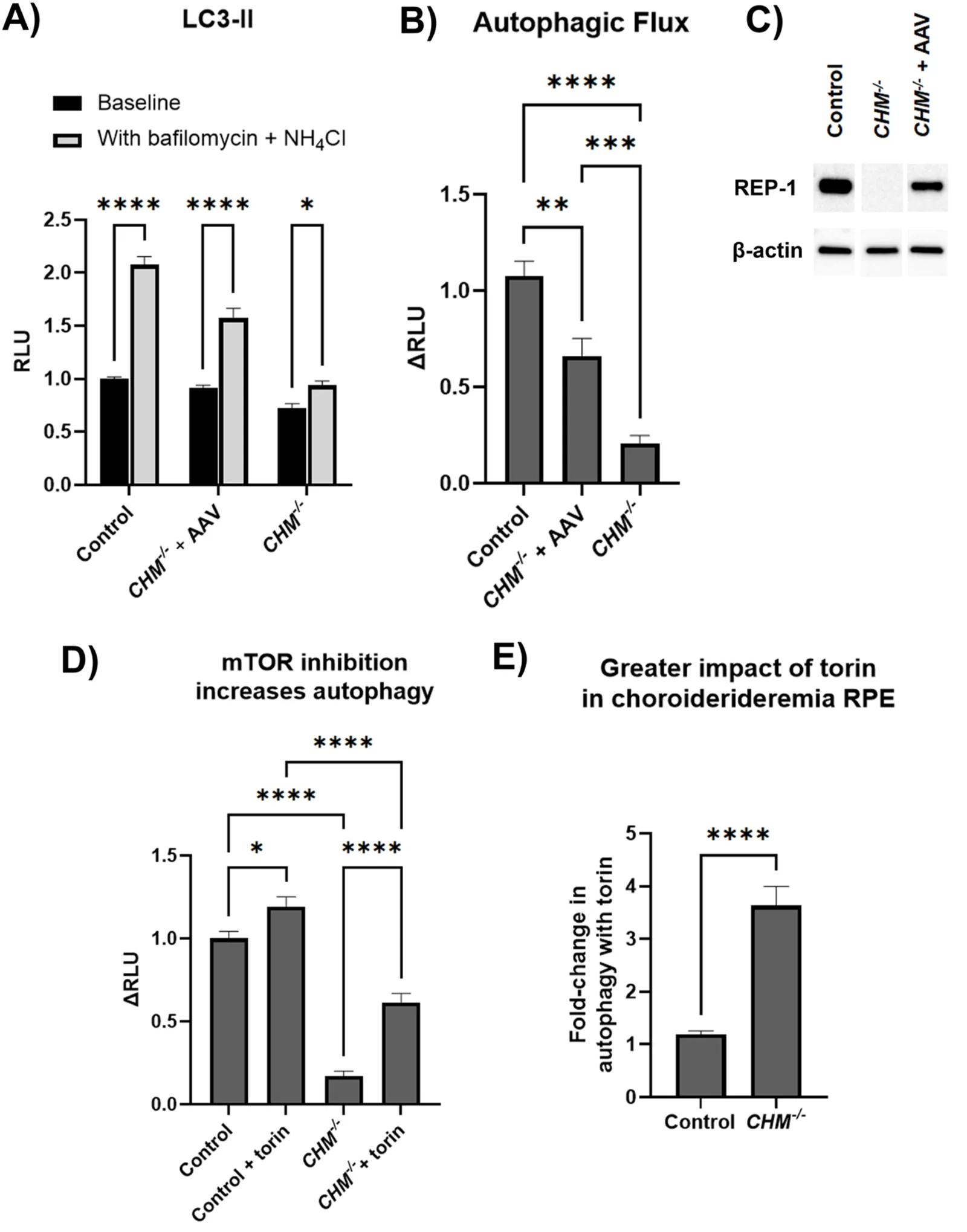
*CHM^−/−^* iPSC-RPE cells have reduced autophagic flux due to mTOR signaling. (**A**) LC3-II, measured by ELISA, increased after autophagic inhibition with bafilomycin (1 µM) and NH_4_Cl (50 mM) for 90 min. (**B**) The difference in LC3-II with and without autophagy inhibition, a.k.a. autophagic flux, was reduced in *CHM^−/−^* iPSC-RPE cells, with partial rescue after gene replacement with ShH10-CMV-*CHM* (AAV-*CHM*). As a control, shH10-CMV-GFP did not impact autophagy (Figure S3). (**C**) Western blot of Rab escort protein 1(REP-1) showing expression in *CHM^−/−^* iPSC-RPE cells 2 weeks post-transduction with 100 K MOI AAV-*CHM*, at approximately 51% of control levels when normalized to β-actin. (**D**) Cells were treated with 0.5 µM of torin for 24 h to inhibit mTOR, which increased autophagic flux in both control and *CHM^−/−^* iPSC-RPE cells. Figure S5 shows the effective inhibition of mTOR with 0.5 µM Torin. (**E**) The fold-change in autophagic flux with torin compared to baseline was greater in *CHM^−/−^* cells (3.6) than control cells (1.2). For all graphs, N = 9, mean ± SEM, 2-way ANOVA (**A**,**D**), 1-way ANOVA (**B**), and Student’s *t*-test (**E**), * *p* < 0.05, ** *p* < 0.01, *** *p* < 0.001,**** *p* < 0.0001. RLU= relative light units, normalized to control baseline value. Experiments in 5A were performed 3 times; experiments in 5D were performed twice.

Autophagic flux was measured in *CHM^−/−^* iPSC-RPE cells by ELISA of autophagosome marker LC3-II with and without bafilomycin and NH_4_Cl (Figure 5A,B). LC3-II is degraded by autophagy and increases with autophagy inhibition with bafilomycin and NH_4_Cl (Figure 5A). The difference in LC3-II with and without autophagy inhibition (a.k.a. autophagic flux) was reduced to 19% of control levels in *CHM^−/−^* iPSC-RPE cells (*p* < 0.0001) (Figure 5B). *CHM^−/−^* iPSC-RPE cells were then transduced with a gene replacement AAV vector encoding *CHM* downstream of a CMV promoter packaged into ShH10, a serotype of AAV2 (ShH10-CMV-*CHM*). Transduced *CHM^−/−^* iPSC-RPE cells showed REP-1 expression levels approximately 51% of control levels when quantified by densitometry (Figure 5C). Transduction with ShH10-CMV-*CHM* increased autophagic flux 3-fold, resulting in a 48% rescue of the autophagic deficit in *CHM^−/−^* iPSC-RPE cells (*p* < 0.001) (Figure 5B).

Torin was used to inhibit mTOR in control and *CHM^−/−^* iPSC-RPE cells. Inhibition with 0.5 µM torin for 24 h was found to reduce mTORC1 signaling in *CHM^−/−^* iPSC-RPE cells to levels approximately equal to or less than control cells (Figure S5). Torin increased autophagic flux in *CHM^−/−^* iPSC-RPE more than control iPSC-RPE cells (3.6-fold vs. 1.2-fold, *p* < 0.0001), thus supporting the role of mTOR signaling in reducing autophagic flux in choroideremia (Figure 5D,E).

### 3.6. Rab12-Deficient Control iPSC-RPE Cells Reproduce the Increased mTORC1 and Reduced Autophagy Flux Observed in CHM^−/−^ iPSC-RPE Cells

**Figure 6:**
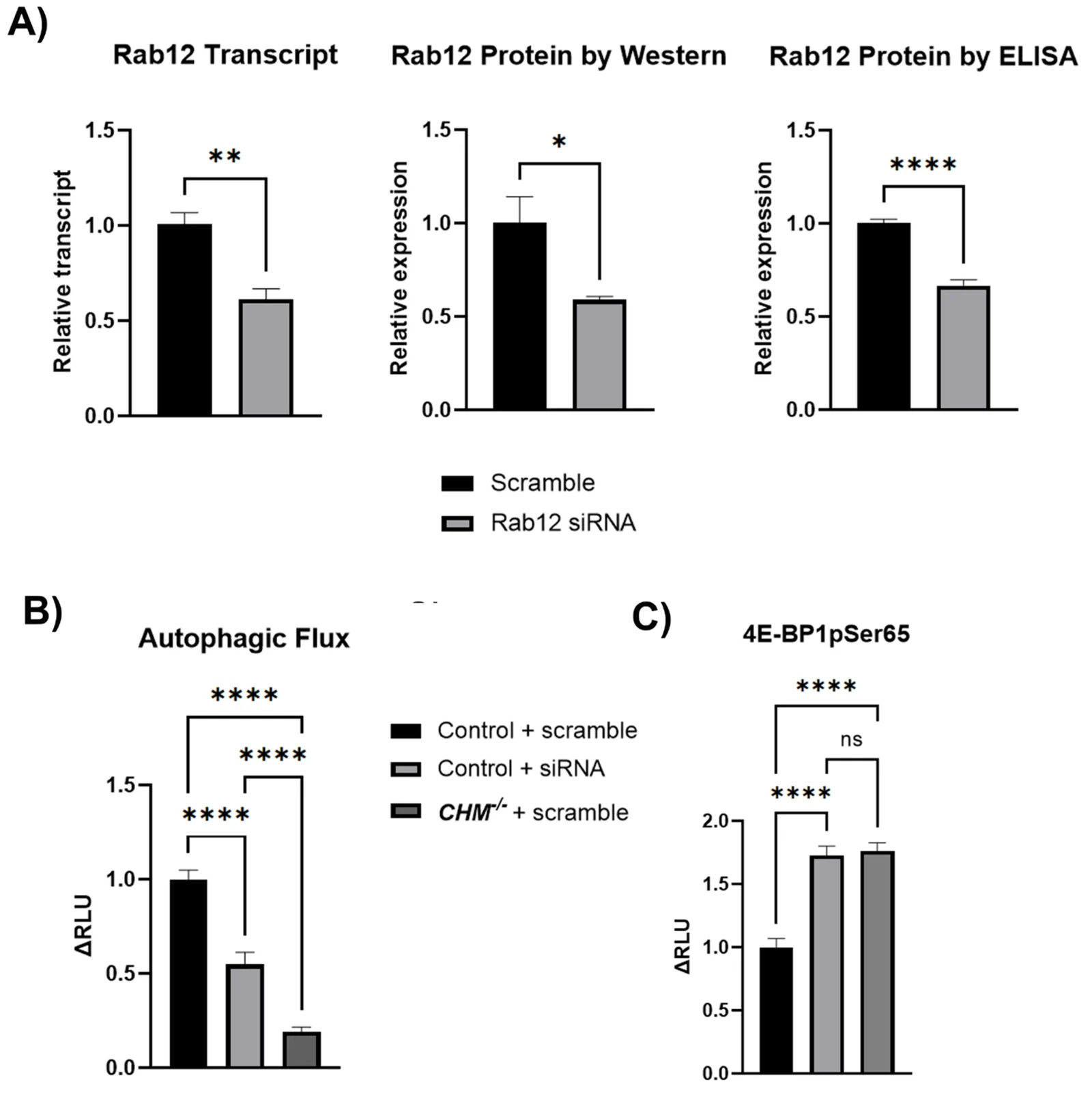
Rab12 knockdown reproduces the increased mTORC1 signaling and reduced autophagic flux observed in *CHM^−/−^* iPSC-RPE cells. (**A**) Pooled siRNA targeting Rab12 were transfected in control iPSC-RPE cells, resulting in a 40% knockdown of Rab12 transcript (n = 3; experiment performed twice), a 41% knockdown of Rab12 protein by western blot (n = 4; experiment performed once), and a 33% knockdown of Rab12 protein by ELISA (n = 38; experiment performed once), compared to cells treated with a scrambled negative control siRNA pool. The western blot of Rab12 protein is in Figure S4. (**B**) Autophagic flux, measured as in Figure 5, was reduced by 45% in control cells treated with Rab12 siRNA and 81% in *CHM^−/−^* cells (treated with scramble siRNA), compared to control cells treated with scramble siRNA (n = 9; experiment performed once). (**C**) Levels of 4E-BP1 phosphorylated on serine 65 were measured by ELISA and used as a marker of mTORC1 activity and were increased 73% in control cells treated with Rab12 siRNA and 76% in *CHM^−/−^* cells (treated with scramble siRNA), compared to control cells treated with scramble siRNA (n = 6; experiment performed once). All graphs show mean ± SEM. Student’s *t*-test was used for (**A**), and one-way ANOVA was used for (**B**,**C**). * *p* < 0.05, ** *p* < 0.01, **** *p* < 0.0001, ns = not significant. RLU = relative light units, normalized to control value.

Pooled siRNA was used to knockdown Rab12 in control iPSC-RPE cells, using scrambled siRNA as a negative control, and a 40% knockdown of Rab12 transcript was achieved (*p* < 0.01). Rab12 protein was knocked down 41% measured by western blot and 33% measured by ELISA (*p* < 0.05 and *p* < 0.0001) (Figure 6A). This level of Rab12 knockdown resulted in a 45% reduction in autophagic flux (*p* < 0.0001), compared to an 81% reduction resulting from *CHM* knockout (*p* < 0.0001), thus reproducing approximately half of the effect seen with *CHM* knockout (Figure 6B). Furthermore, mTORC1 activity was assessed by levels of 4E-BP1 phosphorylated on serine 65 (4E-BP1pSer65), quantified by ELISA. 4E-BP1 is a translational repressor that is inhibited by mTORC1 via phosphorylation [35]. Similar to other mTORC1 targets shown in Figure 4, 4E-BP1pSer65 was significantly higher in in *CHM^−/−^* iPSC-RPE compared to control cells (76% higher, *p* < 0.0001, Figure 6C). Rab12 knockdown in control cells resulted in a 72% increase in 4E-BP1pSer65 (*p* < 0.0001, Figure 6C), thus mimicking the *CHM^−/−^* phenotype.

## 4. Discussion

In this study, we generated a *CHM^−/−^* iPSC-RPE model of choroideremia with isogenic control iPSC-RPE cells for comparison. We used compactin as an unbiased method of global prenylation inhibition to infer the most prenylated and least prenylated Rabs in *CHM^−/−^* iPSC-RPE cells. We demonstrated that mTORC1 signaling is increased, and autophagy is decreased in *CHM^−/−^* iPSC-RPE cells, consistent with loss of function of Rab12, one of the least prenylated Rabs in our model. Furthermore, partial knockdown of Rab12 in control iPSC-RPE cells reproduced the increased mTORC1 signaling and reduced the autophagy phenotype seen with *CHM* knockout, further supporting the importance of Rab12 in RPE autophagic dysfunction in choroideremia.

The *CHM^−/−^* iPSC-RPE cells in this study had *CHM* transcript but no detectable protein. Although stop codons in the non-terminal exon typically result in nonsense-mediated decay (NMD) of the mRNA transcript, it is well established that some genes escape NMD. Choroideremia patients with nonsense variants or frameshift variants and early stop codons have been reported with detectable *CHM* transcript in blood, fibroblasts, and iPSC-RPE cells [29,30]. Based on the early exon 1 frameshift variants in our cell line, the translated REP-1 protein from the two alleles would be expected to have either the first five amino acids followed by a stop codon or the first seven amino acids followed by four incorrect amino acids and a stop codon. These proteins may be unstable and degraded, but even if these peptides persist, they would be unlikely to function or to be detected by a REP-1 antibody. The antibody used in this study was a monoclonal antibody raised against recombinant human REP-1, so it is unknown which domain the antibody recognizes.

Two prior studies have utilized mass spectrometry to comprehensively analyze Rab prenylation in different models of prenylation deficiency, neither using RPE cells. The first used compactin to inhibit prenyl synthesis and globally reduce protein prenylation in HeLa cells. Then exogenous prenyl groups were provided, and cell lysates were collected at different time points to compare how quickly different Rabs are prenylated [15]. They found that Rab38, Rab27a, Rab27b, and Rab42 are prenylated the slowest and concluded that this finding may implicate them in choroideremia, since they may be more impacted by REP-1 deficiency when only REP-2 is present. The second study used *CHM^-/y^* mouse embryonic fibroblasts (MEFs) and labeled prenylated proteins with click chemistry to identify levels of prenylated Rabs compared to isogenic control MEFs [16]. They found that Rab12 was the least prenylated by far in *CHM^-/y^* cells, at 10% of wild-type levels, consistent with our findings in human *CHM^−/−^* iPSC-RPE cells that Rab12 is the least prenylated and further supporting the role of Rab12 in choroideremia. 

A previous siRNA screen of Rabs in mouse embryonic fibroblasts identified Rab12 as a regulator of mTORC1 [32]. The study further showed that Rab12 traffics the amino acid transporter PAT4 to the lysosome for degradation, and that overexpression of PAT4 mimics Rab12 knockdown by activating mTORC1. The authors therefore propose that Rab12 regulates mTORC1 by promoting PAT4 degradation and reducing intracellular amino acids. Rab12 is also known to traffic the transferrin receptor (TFR) for degradation, and iron also has a known role in activating mTORC1 [36,37,38]. Therefore, future investigations are needed to the determine the relative contributions of PAT4 and amino acids vs. TFR and iron to Rab12-mediated mTORC1 signaling in choroideremia RPE cells.

Limitations of this study include the use of RPE cell culture alone without in vivo validation. Patient histology, in vivo imaging studies, and animal models all demonstrate that primary RPE pathology contributes significantly to choroideremia [2,3,4,5]. However, the mouse model provides evidence for both primary RPE cell and photoreceptor pathology contributing to retinal degeneration [6]. Furthermore, the impact of RPE cell dysfunction on chorioretinal degeneration cannot be fully understood without future studies validating the cell culture findings in an in vivo model to account for the complex intercellular signaling between RPE cells, photoreceptors, and the choroid. Another limitation is the lack of data on the most unprenylated Rabs in control cells for comparison, although nearly all Rabs in control cells are prenylated as seen in Figure 3B, diminishing the significance of this limitation. Though TMT spectrometry was not feasible in the control cells due to the scarcity of unprenylated Rabs for streptavidin pull-down, our validation studies showing western blot of membrane-bound Rabs 12, 27a, 8a, and 5a used both *CHM^−/−^* and control iPSC-RPE cells. These four Rabs were chosen to represent the spectrum of Rabs in Figure 3C, including two of the least prenylated (Rabs 12 and 27a), one of the most prenylated (Rab5a), and one in the middle (Rab8a). Western blot is semi-quantitative, and though Rabs8a and 5a looked similar in their membrane distribution using this method, the overall order of prenylation status among the four Rabs was similar to the results obtained from TMT spectrometry.

Rab27a was the first Rab reported to be unprenylated in choroideremia, which has since been validated in multiple reports, including the present study [9,14,15,39]. Rab27a plays a role in exosome secretion and melanosome transport and is abundant in pigmented cells and cells with high secretory load, both characteristics of RPE cells [40,41,42,43]. Despite interest in Rab27a in choroideremia, a molecular mechanism linking Rab27a under-prenylation with chorioretinal degeneration has not been elucidated. Autosomal recessive loss-of-function variants in Rab27a cause Griscelli Syndrome, characterized by partial albinism, neurologic deficits, and immunodeficiency secondary to defective lymphocyte granule exocytosis (OMIM#607624). Of note, retinal degeneration has not been reported in these patients, although it cannot be ruled out due to high childhood mortality [44].

Previous cell culture models of choroideremia have implicated lysosome dysfunction in disease pathogenesis [14,23]. *CHM*-deficient RPE cells have demonstrated delayed degradation of phagocytosed outer segments and reduced acidification of phagosomes. Lysosomes are intimately linked with autophagy, which requires fusion of the lysosome and autophagosome to digest waste, and downregulation of both lysosomal genes and autophagy genes are downstream of the mTORC1 signaling transcriptional program [45]. Thus, our findings of increased mTORC1 signaling and reduced autophagy are consistent with these previous reports. Defective RPE autophagy is also a well-established mechanism of disease in age-related macular degeneration (AMD) in numerous published models, as well as Stargardt Disease and PROM1-associated retinal degeneration [18,19,20,21,22]. Activation of autophagy reduced lipofuscin accumulation in RPE cell culture and improved retinal function and reduced Bruch’s membrane thickening in an APOE-deficient mouse model of AMD [19,20]. In the *Abca4^−/−^* mouse model of Stargardt disease, increased RPE cell endocytosis of complement factor C3a resulted in increased mTOR activation [21]. Similarly, knockout of PROM1 in both human and mouse RPE cells led to increased mTORC1 activity and reduced autophagy [22,46]. Autophagy is a therapeutic target in pre-clinical studies and in an ongoing clinical trial for Stargardt Disease [NCT04545736]. Defective autophagy may therefore be a shared pathway contributing to multiple forms of chorioretinal degeneration.

In summary, this study defines the spectrum of unprenylated Rabs in the tissue of interest in choroideremia using a human RPE model and further demonstrates downstream consequences of unprenylated Rab12, one of the most severely unprenylated Rabs in our study. The role of Rab12 is supported by a prior study in which it was also the most severely unprenylated in a murine cell culture model and is further substantiated in the present study by the downstream consequences of increased mTORC1 signaling and reduced autophagy, which are known to play a role in other chorioretinal degenerative diseases. RPE autophagy, or upstream mTORC1 signaling or Rab12 prenylation, may therefore warrant further investigation as a promising future therapeutic target to save vision in choroideremia.

## Data Availability

Mass spectrometry data are deposited in Deep Blue Data at the following link: https://doi.org/10.7302/hyxk-9388.

## Supplementary Materials

The following supporting information can be downloaded at: https://www.mdpi.com/article/10.3390/cells13121068/s1, Figure S1: Timeline of iPSC-RPE differentiation; Figure S2: Expression of NaK ATPase is equivalent in *CHM^−/−^* and control iPSC-RPE cells; Figure S3: AAV does not impact autophagy. Figure S4: Western blot of Rab12 and β-actin loading control with Rab12 siRNA knockdown compared to scramble negative control siRNA in control iPSC-RPE cells; Figure S5: Torin inhibition of mTORC1 signaling. Table S1: Primer sequences for RT-PCR.
