# Correction: Raeker et al. Reduced Retinal Pigment Epithelial Autophagy Due to Loss of Rab12 Prenylation in a Human iPSC-RPE Model of Choroideremia. *Cells* 2024, *13*, 1068

**DOI:** 10.3390/cells15141303

**Published:** 2026-07-21

**Authors:** Maide Ö. Raeker, Nirosha D. Perera, Athanasios J. Karoukis, Lisheng Chen, Kecia L. Feathers, Robin R. Ali, Debra A. Thompson, Abigail T. Fahim

**Affiliations:** 1 Department of Ophthalmology, University of Michigan, Ann Arbor, MI 48105, USA; mraeker@med.umich.edu (M.Ö.R.); nperera@med.umich.edu (N.D.P.); akarouki@med.umich.edu (A.J.K.); keciaf@med.umich.edu (K.L.F.); robin.ali@kcl.ac.uk (R.R.A.); dathom@med.umich.edu (D.A.T.); 2 Department of Orthopedic Surgery, University of Michigan, Ann Arbor, MI 48109, USA; lishengc@med.umich.edu; 3 KCL Center for Cell and Gene Therapy, London WC2R 2LS, UK; 4 Department of Biological Chemistry, University of Michigan, Ann Arbor, MI 48109, USA

## Missing Citation

In the original publication [1], “**[24]**” was not cited in **Figure 1**. The citation has now been inserted in “**3. Results**”, “**3.1. CHM^−/−^ iPSC-RPE Cells Were Generated**”, “**Figure 1 Legend**” and should read:

**Figure 1.** Generation of *CHM^−/−^* iPSC-RPE cells. (**A**) CRISPR/Cas-9 knockout of *CHM* targeting exon 1, resulting in biallelic variants with early stop codons (*). Sanger sequencing of the biallelic deletions is shown. (**B**) Western blot showing absence of Rab escort protein 1 in knockout (KO) iPS cells compared to wild-type (WT) iPS cells (representative blot shown; experiment was performed 3 times); and (**C**) KO and WT iPSC-RPE cells with immunocytochemistry (ICC) showing absence of Rab escort protein in KO cells (representative image shown; experiment was performed twice). A magnified image of iPSC-RPE cobblestone morphology is shown at bottom right and has been shown previously [24].

The authors state that the scientific conclusions are unaffected. This correction was approved by the Academic Editor. The original publication has also been updated.
