# Supplementary material for: Reduced Retinal Pigment Epithelial Autophagy Due to Loss of Rab12 Prenylation in a Human iPSC-RPE Model of Choroideremia
Source: Cells. 2024 Jun 19;13(12):1068. doi: 10.3390/cells13121068 (PMC11201631; doi:10.3390/cells13121068)
Supplement: Supplementary file 1 [file cells-13-01068-s001.zip › cells-3003356-supplementary.pdf]

Supplemental Materials

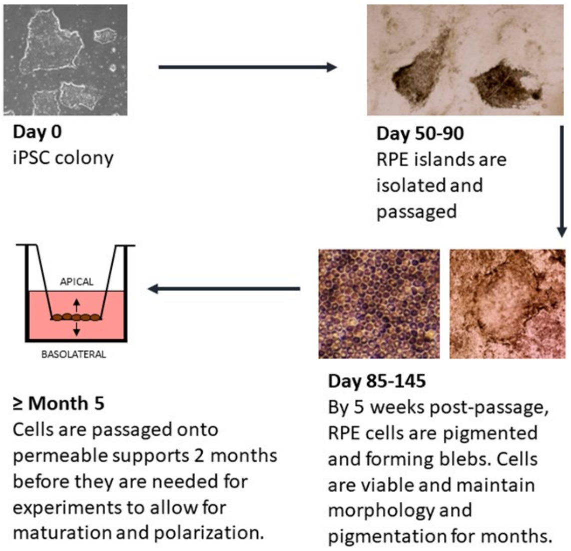

Figure S1. Timeline of iPSC-RPE differentiation. The timeline of the iPSC-RPE differentiation protocol used in this manuscript is shown.

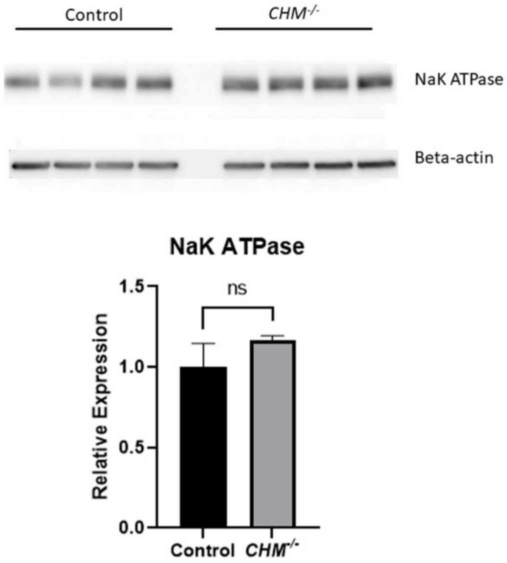

Figure S2. Expression of NaK ATPase is equivalent in *CHM*$^{-/-}$ and control iPSC-RPE cells. Western blot for NaK ATPase in cell lysates. Densitometry was normalized to beta actin and shown in the graph, n=4, mean±SEM, with no significant difference by student T test, experiment performed once.

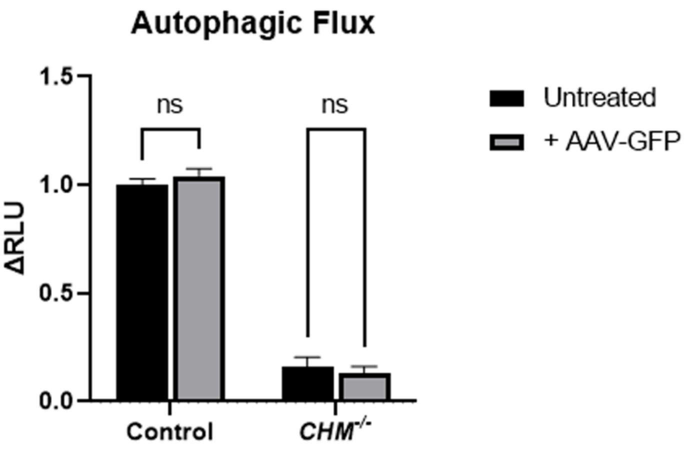

Figure S3. AAV does not impact autophagy. The difference in LC3-II by ELISA with and without autophagic inhibition with bafilomycin and NH_4Cl (a.k.a. autophagic flux) is graphed for control and *CHM*$^{-/-}$ iPSC-RPE cells, with and without treatment with a serotype of AAV containing the GFP gene (shH10-CMV-GFP). Power calculations of Cohen's D indicated a sample size of 20 gives 80% power to detect a 20% difference between groups. N=20, mean±SEM, 2-way ANOVA, experiment performed once. RLU= relative light units, normalized to control baseline value.

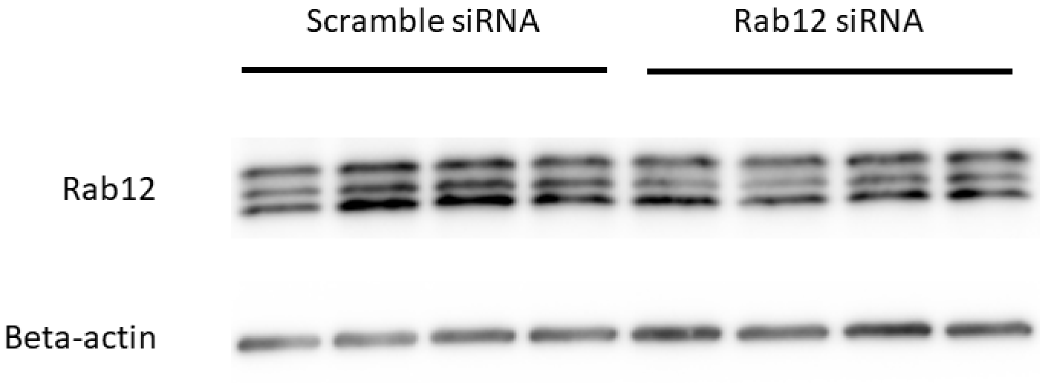

Figure S4. Western blot of Rab12 and β-actin loading control with Rab12 siRNA knockdown compared to scramble negative control siRNA in control iPSC-RPE cells. Densitometry of Rab12 normalized to β-actin was used for the graph in Figure 6A. Experiment performed once.

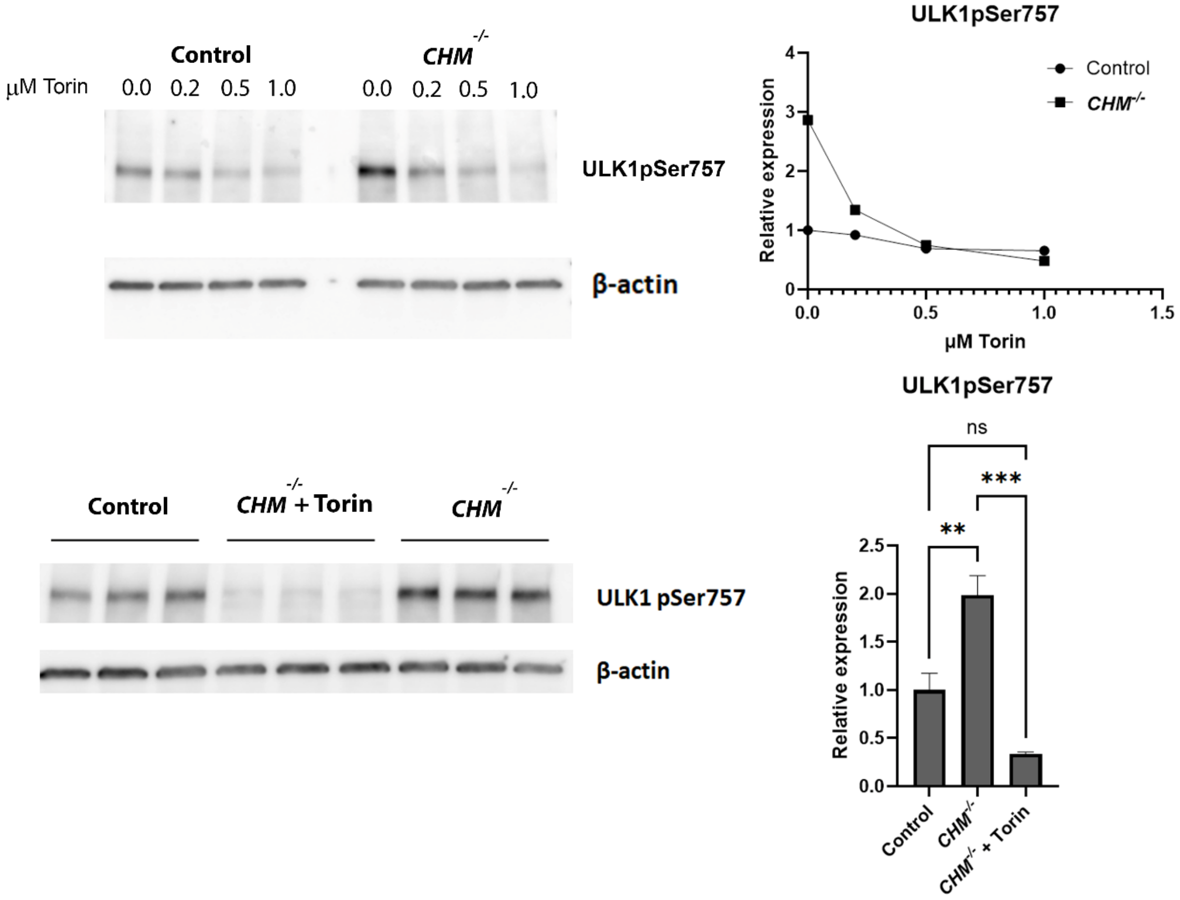

Figure S5. Torin inhibition of mTORC1 signaling. Control and *CHM*$^{-/-}$ iPSC-RPE cells were treated with different concentrations of Torin. Expression of ULKpSer757 as a marker of mTORC1 activity was measured by western blot (upper left). A graph of densitometries from the western blot showing relative expression of ULKpSer757 normalized to Beta-actin is on the upper right. Triplicate samples of *CHM*$^{-/-}$ iPSC-RPE cells were then treated with 0.5 uM Torin and ULKpSer757 levels were compared to untreated *CHM*$^{-/-}$ and control iPSC-RPE cells in the western blot on the lower left. Densitometries are graphed on the lower right. Mean ±SEM, One-way ANOVA, **p<0.01, ***p<0.001. Experiment performed once.

Table S1. Primer sequences for RT-PCR

**Gene**	**Forward**	**Reverse**
CHM	CCGGAGAGAGTTCTGCATGTTG	AAGGATCTGGTCTTGCCACA
CHML	AGAATTCTGCCCTCCACCTC	ATGCCATGAAGGAGGTCCAA
RAB12	ACAGAGAAATCACCAGGCAG	TCTCGTCCACATTGAAGTTATCC
ACTB	CAGGATGCAGAAGGAGATCAC	TGTCAAGAAAGGGTGTAACGC
GAPDH	CTGGGCTACACTGAGCACC	AAGTGGTCGTTGAGGGCAA
